# Antimicrobial Peptide Brevivin-1RL1 from Frog Skin Secretion Induces Apoptosis and Necrosis of Tumor Cells

**DOI:** 10.3390/molecules26072059

**Published:** 2021-04-03

**Authors:** Xiaoman Ju, Dongmei Fan, Lingmei Kong, Qihong Yang, Yiying Zhu, Shaohua Zhang, Guifeng Su, Yan Li

**Affiliations:** 1 State Key Laboratory of Phytochemistry and Plant Resources in West China, Kunming Institute of Botany, Chinese Academy of Sciences, Kunming 650201, China; juxiaoman@mail.kib.ac.cn (X.J.); fandongmei@mail.kib.ac.cn (D.F.); konglingmei@mail.kib.ac.cn (L.K.); yangqihong@mail.kib.ac.cn (Q.Y.); zhuyiying@mail.kib.ac.cn (Y.Z.); zhangshaohua@mail.kib.ac.cn (S.Z.); suguifeng@mail.kib.ac.cn (G.S.); 2 University of Chinese Academy of Sciences, Beijing 100049, China; 3 Yunnan Key Laboratory of Natural Medicinal Chemistry, Kunming Institute of Botany, Chinese Academy of Sciences, Kunming 650201, China

**Keywords:** antimicrobial peptide, Brevivin-1RL1, apoptosis, necrosis, caspase, anticancer

## Abstract

Cancer has always been one of the most common malignant diseases in the world. Therefore, there is an urgent need to find potent agents with selective antitumor activity against cancer cells. It has been reported that antimicrobial peptides (AMPs) can selectively target tumor cells. In this study, we focused on the anti-tumor activity and mechanism of Brevivin-1RL1, a cationic α-helical AMP isolated from frog *Rana limnocharis* skin secretions. We found that Brevivin-1RL1 preferentially inhibits tumor cells rather than non-tumor cells with slight hemolytic activity. Cell viability assay demonstrated the intermolecular disulfide bridge contributes to the inhibitory activity of the peptide as the antitumor activity was abolished when the disulfide bridge reduced. Further mechanism studies revealed that both necrosis and apoptosis are involved in Brevivin-1RL1 mediated tumor cells death. Moreover, Brevivin-1RL1 induced extrinsic and mitochondria intrinsic apoptosis is caspases dependent, as the pan-caspase inhibitor z-VAD-FMK rescued Brevinin-1RL1 induced tumor cell proliferative inhibition. Immunohistology staining showed Brevivin-1RL1 mainly aggregated on the surface of the tumor cells. These results together suggested that Brevivin-1RL1 preferentially converges on the cancer cells to trigger necrosis and caspase-dependent apoptosis and Brevivin-1RL1 could be considered as a pharmacological candidate for further development as anti-cancer agent.

## 1. Introduction

Although great progress has been made in the treatment of cancer in recent years [1,2,3], traditional therapies still have many defects and problems, such as widespread side effects due to non-adequate specificity for tumor cells and the tendency to develop multiple drug resistance, which greatly limit their therapeutic efficacy [4,5]. As a result, it is urgent to develop potent agents with selective antitumor activity against cancer cells. 

Antimicrobial peptides (AMPs), also referred to as host defense peptides, have been isolated and characterized from a wide variety of organisms, such as microorganisms, insects, plants, birds, fish, amphibians and mammals, including humans, where they play an important role in the innate defense against microbial and viral invasions [6,7]. AMPs have a wide spectrum killing activity including Gram-positive and Gram-negative bacteria, fungi and protozoa without cytotoxicity towards normal mammal cells at the effective dose [8]. AMPs were initially discovered due to their role in the clearance of microorganisms [9,10]. As more natural-source peptides have been discovered, other biological activities of these peptides have been explored, such as anti-fungal, anti-viral and immune regulation, as well as anti-cancer activities [8,11]. According to the record, there are 3180 kinds of AMPs in the current Antimicrobial Peptide Database (APD), among which 237 peptides with anti-tumor activity are included. 

Numerous naturally derived AMPs inhibit cancer cells mainly through plasma membrane disruption or non-membranolytic cytotoxicity [12]. AMPs, such as Defensin-peptides A, B [13], NK-2 [14] MPI-1 [15], Melittin [16], Pardaxin [17] and magainin 2 (MG2) [18], target and form pores on the cancer cell membranes to attenuate cancer cells, but Melittin is also toxic to non-cancer cells and has a powerful hemolytic activity. Non-membranolytic pathway mainly involves reactive oxygen species, DNA damage response [19], immune regulation [20], autophagy and apoptosis [21]. For example, FK-16 derived from LL-37 could induce tumor cell death by activating non-caspase-dependent apoptosis and autophagy [22]. LZ1, derived from the snake venom cathelicidin, could inhibit the activity of mTORC1 by targeting degradation of cell surface-expressed nucleolin to activate AMPK, thereby inhibiting the production of autophagic flux [23]. Hence, the current research on AMPs indicated that they might be potential drugs for future cancer treatment.

Brevivin-1RL1 is a member of the amphibian family of cationicAMPs originally isolated from *Rana limnocharis* composed of 24 amino acids and sharing an intra-molecular disulfide-bridged domain of 7-membered ring located at the C-terminus. It has been shown that Brevivin-1RL1 displays broadspectrum antibacterial activity [24]. In the present study, we disclosed that Brevivin-1RL1 preferential cytotoxicity for tumor cells to induce necrosis and caspase-dependent apoptosis to suppress tumor growth. Our study not only revealed the application of Brevivin-1RL1 as a potential candidate antitumor agent, but also further confirmed the possibility of AMPs as a promising anticancer drug.

## 2. Results

### 2.1. Synthesis, Purification and Characterization of Peptides

**Figure 1:**
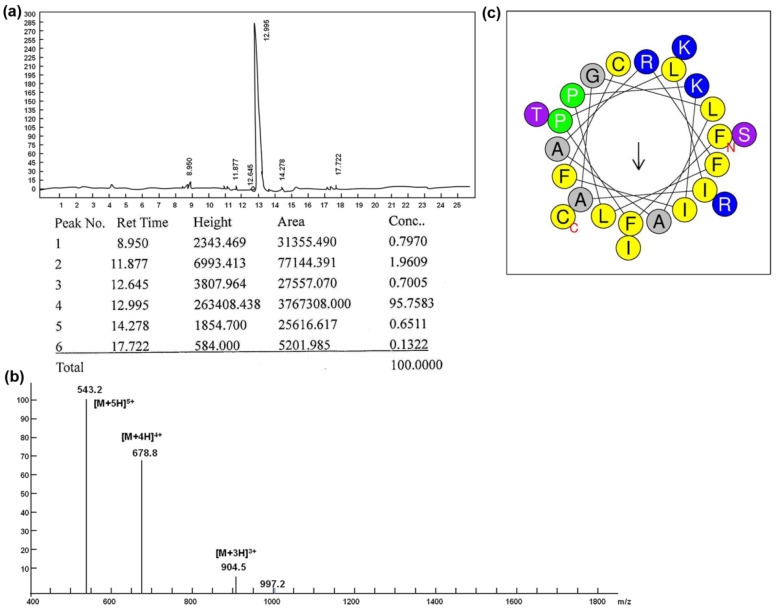
The purity, mass spectrometry and structure of Brevinin-1RL1. (**a**) The HPLC chromatogram of Brevinin-1RL1. (**b**) The mass spectrometry (MS) of Brevinin-1RL1. (**c**) Helical wheel projection of Brevinin-1RL1. The helical wheel arrangement of Brevinin-1RL1 was produced using the HeliQuest website (http://heliquest.ipmc.cnrs.fr/) (accessed on: 12 July 2020). The red N represents the N-terminal of the peptide sequence. The red C represents the C-terminal of the peptide sequence. Arrow indicates direction of the hydrophobic moment.

Brevinin-1RL1 is composed of 24 amino acids and it contains an intermolecular disulfide bridge consisting of seven amino acid residues at the C-terminus of the peptide. Table 1 shows the primary sequence and other biophysical parameters. The peptide was synthesized and purified by reverse-phase HPLC with the purity 95% (Figure 1a, b). In addition, the HPLC chromatogram and MS of FITC labeled Brevinin-1RL1 are shown in Supplementary Materials Figure S1a,b, respectively. The helical wheel arrangement of Brevinin-1RL1 was produced using the HeliQuest website (http://heliquest.ipmc.cnrs.fr/) (accessed on: 12 July 2020) and the result demonstrated that Brevinin-1RL1 adopted an α-helix structure with a hydrophobic face consisting of I, A, F, I, L, A, C, F and A (Figure 1c).

### 2.2. Brevinin-1RL1 Displays Cytotoxicity towards Tumor Cells with Moderate Hemolysis

**Figure 2:**
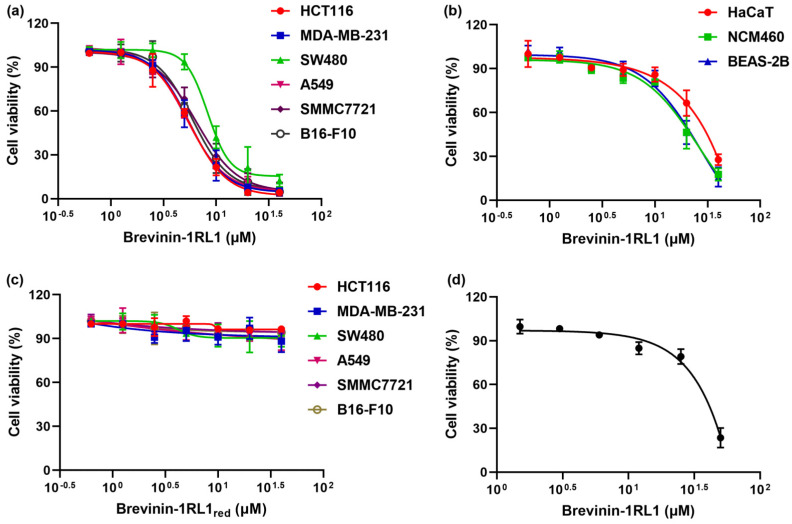
Brevinin-1RL1 preferentially suppressed cancer cells. The cell viability of Brevinin-1RL1 on different tumor cell lines (HCT116, MDA-MB-231, SW480, A549, SMMC7721, B16-F10) (**a**) and three non-tumor cell lines (HaCaT, NCM460 and BEAS-2B) (**b**) were determined by MTS assay. Tumor cells seeded in 96-well plates were incubated with various concentrations of Brevinin-1RL1 for 48 h and the cell viability was measured by MTS. (**c**) The cell viability of tumor cell lines treated with various concentrations of Brevinin-1RL1_red_ was measured. (**d**) The erythrocyte viability of Brevinin-1RL1 on human erythrocytes was detected by hemolytic activity. Equal volume of Brevinin-1RL1 and human erythrocytes were incubated for 30 min, the absorbance of the supernatant was measured at 540 nm. Each experimental data is three independent experiments.

To explore the antitumor activity and selectivity profile of Brevinin-1RL1, cell viability assay was conducted in six human cancer cell lines and three noncancer cell lines by 3-(4,5-dimethylthiazol-2-yl)-5-(3-carboxymethoxyphenyl)-2-(4-sulfophenyl)-2H-tetrazolium (MTS) assay. As shown in Figure 2a and Table 2, Brevinin-1RL1 showed a dose-dependent inhibitory effect on various tumor cell lines (HCT116, MDA-MB-231, SW480, A549, SMMC-7721, B16-F10) with the IC_50_ values ranging from 5 to 10 μM (Table 2). Of note, it was less toxic to noncancer cell lines, including human noncancer colonic epithelial cell line NCM460, human noncancer bronchus epithelial cell line BEAS-2B and human keratinocyte cell line HaCaT, especially in the human keratinocyte cell line HaCaT (28.67 μM) (Figure 2b). The toxicity of Brevinin-1RL1 toward cancer cells was generally about 3 times higher than toward the noncancer cell lines, indicating Brevinin-1RL1 preferentially suppressed cancer cell lines. 

The Rana-box disulfide motif is a unique structure and mainly found in antimicrobial peptides from frogs, which might contribute to the functional activity of antimicrobial peptides [25]. Here, we explored the proliferation inhibitory activity of Brevinin-1RL1 with the disulfide reduced (Brevinin-1RL1_red_). As shown in Figure 2c, the antitumor activity the peptide was deprived when the “Rana box” structure was removed. The result indicated that the structure of “Rana box” might be essential for maintaining the biological potency of Brevinin-1RL1. Furthermore, no serious hemolysis was detected in the human erythrocytes treated with Brevinin-1RL1 at the IC_50_ of cancer cells and only about 30% hemolysis was observed at 4–6-fold higher concentrations than IC_50_ (Figure 2d). These results suggested that Brevinin-1RL1 had a certainly selectivity to tumor cells and its hemolytic activity was low at the effective concentration. 

### 2.3. Brevinin-1RL1 Induces Cell Apoptosis and Necrosis

**Figure 3:**
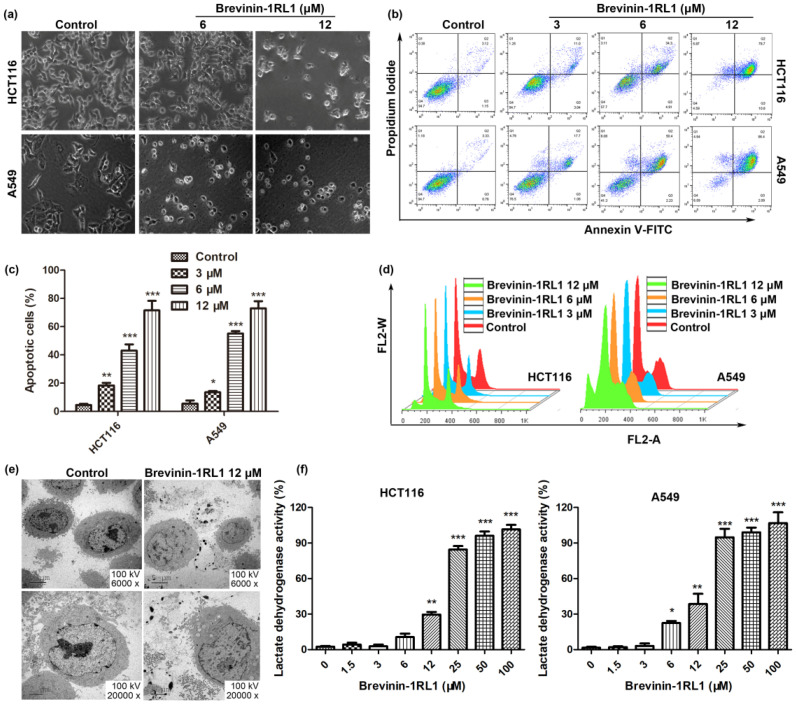
Brevinin-1RL1 induced cell apoptosis and necrosis in A549 and HCT116 cells. (**a**) The effect of Brevinin-1RL1 on morphological alteration of the tumor cell lines HCT116 and A549 cells was observed with Olympus microscope (Olympus Optical Co., Melville, NY). (**b**) Brevinin-1RL1 dose-dependently induced apoptosis of tumor cells. HCT116 and A549 cells were seeded in 6-well plates and co-incubated with different concentrations of Brevinin-1RL1 for 48 h. Apoptosis was then detected by flow cytometry using Annexin-V and PI double staining. (**c**) The apoptotic percentage of cells was statistically analyzed. (**d**) The distribution of cell cycle was detected by flow cytometry in Brevinin-1RL1 treated cells. HCT116 and A549 cells inoculated in 6-well plates were treated with indicated concentrations of Brevinin-1RL1 or control for 24 h. The cells were collected for propidium iodide (PI) staining and the cell cycle distribution was detected by flow cytometry. (**e**) Brevinin-1RL1 induced morphological hallmarks of cell necrosis. HCT116 cells were incubated for 12 h with and without Brevinin-1RL1 and the specific morphology of the cells was observed by TEM. (**f**) Brevinin-1RL1 induced the release of LDH in tumor cells. A549 and HCT116 cells were seeded in 96-well plates with 5 × 10^3^ cells/well/100 μL and incubated with different concentrations of Brevinin-1RL1 for 24 h. Cytoplasmic LDH release of cells was determined by the microplate reader. Each experimental data is three independent experiments. * p < 0.05, ** p < 0.01, *** p < 0.001 in comparison to negative controls, and indicated the significant difference between the two groups. Data expressed as the mean ± SD (*n* = 3).

Brevinin-1RL1 preferential inhibited the proliferation of cancer cells, while the mechanism stayed unclear. Thus, the mechanism Brevinin-1RL1 induced proliferation was further explored. To begin with, the cell morphology changes of cells exposed to Brevinin-1RL1 or equivalent solvent (as control) were observed using optical microscope. As shown in Figure 3a, Brevinin-1RL1 treatment not only reduced the density of HCT116 and A549 in a dose-dependent manner, but also changed the tumor cells’ shape. After treated with Brevinin-1RL1 at 6 μM, some cells displayed cell shrinkage, the sign of apoptosis. In addition, when the cells were incubated with 12 μM Brevinin-1RL1 within 24 h, a large amount of vacuolation occurred and cell debris appeared in the cell supernatant, indicating the occurrence of necrosis. Therefore, we hypothesized that Brevinin-1RL1 inhibition of tumor cells growth may be involved in both apoptosis and necrosis. 

Cell apoptosis is the predominant cell death type, then an Annexin V-fluorescein isothiocyanate (FITC) and Propidium Iodide (PI) double staining assay was undertaken to explore whether Brevinin-1RL1 induced cell apoptosis contributed to the proliferation inhibition. As shown in Figure 3b, an increased percentage of late apoptotic cells (Annexin V+/PI+) were observed in a dose-dependent manner and cell necrosis (PI+) was also detected in cells treated with Brevinin-1RL1. The apoptotic percentages of A549 and HCT116 cells were statistically analyzed as Figure 3c. Moreover, Brevinin-1RL1 resulted in accumulation of HCT116 and A549 cells in the sub-G1 phase representative of apoptotic cells in the PI single staining assay, which further confirmed Brevinin-1RL1 induced apoptosis to inhibit tumor growth. In addition, slight G0/G1-phase arrest was also detected. 

Cell necrosis triggers swelling of cell mitochondria, followed by rupture of the plasma membrane and release of the cytoplasmic contents [26,27]. As shown in Figure 3e, compared with the control group, there were no typical autophagosome structures and acidic vesicles detected in Brevinin-1RL1 treated HCT116 cells, indicating Brevinin-1RL1 induced cell death was autophagy independent, while a large number of vacuoles, cell rupture and release of cytoplasmic contents were all observed in Brevinin-1RL1 treated HCT116 cells with transmission electron microscopy (TEM), which are typical characteristics of cell necrosis. In addition, Brevinin-1RL1 induced cell necrosis was further confirmed by detecting the release of lactate dehydrogenase (LDH) (Figure 3f), a stable cytosolic enzyme released into the cellular environment upon the cell membrane damaged, indicating that Brevinin-1RL1 induced necrosis at high concentrations.

### 2.4. Brevinin-1RL1-Induced Cancer Cells Apoptosis Is Caspase-Dependent 

**Figure 4:**
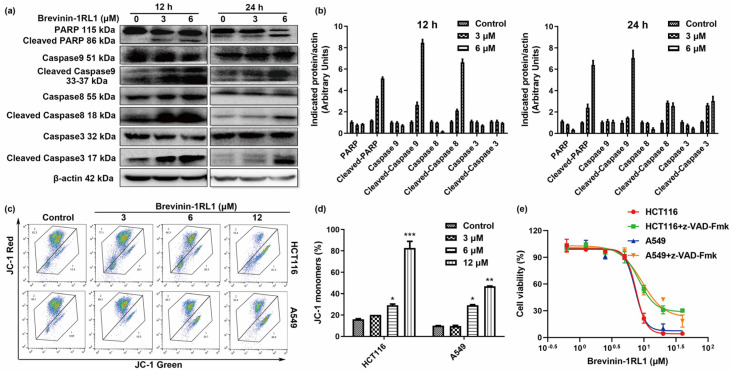
Brevinin-1RL1 induced cell apoptosis is caspase dependent. (**a**) HCT116 cells were incubated with Brevinin-1RL1 for 12 h or 24 h. Cell lysis extracts were analyzed by immunoblotting to detect the cleaved activation of caspase (caspase 3, 8, 9) and its substrates PARP. (**b**) Software Image J 1.52v (National Institutes of Health, Bethesda, USA) was used to analyze the gray value of caspase family proteins. (**c**) Effect of Brevinin-1RL1 on mitochondrial membrane potential. HCT116 and A549 cells were treated with different concentrations of Brevinin-1RL1 for 24 h and subjected to JC-1 stain dye and flow cytometry analysis. When the mitochondrial membrane potential is high, JC-1 aggregates in the matrix of mitochondria to form polymer and shows red fluorescence. However, when the mitochondrial membrane potential is low, JC-1 cannot aggregate in the matrix of mitochondria. At this point, JC-1 is a monomer and shows green fluorescence. The gating of the population was determined according to the change of fluorescence. (**d**) Quantification of the amount of JC-1 monomers and JC-1 aggregates for (**c**). (**e**) The rescuing effect of pan-caspase inhibitor z-VAD-Fmk on Brevinin-1RL1-induced cell death. Cells were preincubated with pan-caspase inhibitor z-VAD-Fmk for 2 h, followed by different concentrations of Brevinin-1RL1 treated for 48 h. The cell viability of cells was determined by MTS assay. * p < 0.05, ** p < 0.01 and *** p < 0.001 in comparison to negative controls, and indicated the significant difference between the two groups for indicated comparison. Data expressed as the mean ± SD (*n* = 3).

Caspases are crucial mediators of apoptosis and regulate extrinsic and intrinsic apoptotic pathways according to the caspases involved. The extrinsic induces the cleavage of caspase 8, while the intrinsic pathway activates caspase 9, leading to the subsequent activation of caspase 3 and poly (ADP-ribose) polymerase (PARP) to induce apoptosis. To verify whether both of the pathways are involved in Brevinin-1RL1 induced apoptosis, the cleavages of caspases were detected. As shown in Figure 4a,b, both the extrinsic apoptosis indicator caspase 8 and the intrinsic caspase 9 were cleaved to activate caspase 3 and PARP. The activation of caspases leads to loss of mitochondrial membrane potential (MMP) [28,29]; thus, the MMP was assessed by the dual-emission potential-sensitive probe JC-1 dye. In intact cells, the JC-1 dye aggregates in the mitochondria and emits red fluorescence, while the dye remains monomers in the cytoplasm and exhibits green fluoresces in apoptotic cells. As shown in Figure 4c,d, Brevinin-1RL1 treatment induced aggregation of monomers, indicating the loss of MMP. These results suggested both the caspases mediating extrinsic and mitochondria intrinsic pathways were activated in Brevinin-1RL1 induced apoptosis. To confirm that Brevinin-1RL1 induced proliferation inhibitory activity is caspase dependent, the cell viabilities of HCT116 and A549 cells treated with Brevinin-1RL1 were measured in the absence or presence of the pan-caspase inhibitor z-VAD-FMK with cell viability assay. Figure 4e showed that z-VAD-FMK effectively rescued Brevinin-1RL1 induced proliferation inhibitory activity, indicating Brevinin-1RL1 induced apoptosis is, at least in part, dependent on caspases mediated extrinsic and mitochondria intrinsic pathways.

### 2.5. Brevinin-1RL1 Aggregates on the Surface of Tumor Cells 

**Figure 5:**
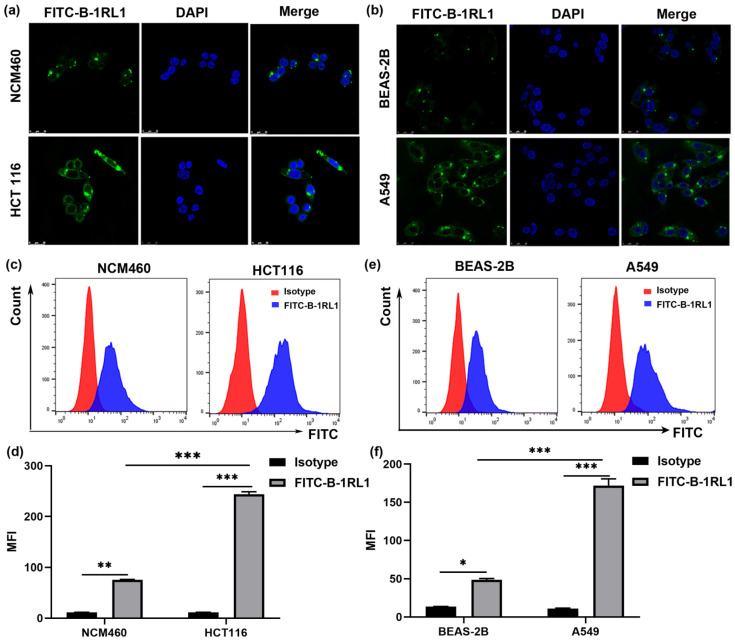
Brevinin-1RL1 localization and binding affinity in tumor cells. Location of Brevinin-1RL1 in NCM460 and HCT116 cells (**a**), BEAS-2B and A549 cells (**b**). Cells were seeded on glass-bottom 6-well plates and treated with FITC-labeled Brevinin-1RL1 for 12 h. Then, the FITC-B-1RL1 was traced and recorded using laser scanning confocal microscopy. (**c**) The binding affinity of Brevinin-1RL1 in NCM460 and HCT116 cells (**c**), BEAS-2B and A549 cells (**e**). 1 × 10^6^/mL HCT116, NCM460, A549 and BEAS-2B cells were collected and incubated with FITC-B-1RL1 or an equal dose of FITC Isotype as controlin the dark for 1 h. Fluorescence alteration was detected by flow cytometry. (**d**) The MFI (Median Fluorescence Intensity) value was statistics from (**c**). (**f**) The MFI value was statistics from (**e**). * p < 0.05, ** p < 0.01 and *** p < 0.001 in comparison to negative controls (Isotype), and indicated the significant difference between the two groups. Data expressed as the mean ± SD (*n* = 3).

In order to investigate the preliminary antitumor mechanism of Brevinin-1RL1, the intracellular localization and binding specificity of Brevinin-1RL1 were conducted with FITC-labeled Brevinin-1RL1. As shown in Figure 5a,b, compared with non-tumor cells NCM460 and BEAS-2B, FITC-labeled Brevinin-1RL1 (FITC-B-1RL1) preferential aggregated on the surface of HCT116 and A549 cells. Meanwhile, to further confirm the affinity of Brevinin-1RL1 to tumor cells, the distribution of FITC-labeled Brevinin-1RL1 on cell surface was also evaluated with flow cytometry and cell surficial accumulation of FITC fluorescence was observed in HCT116 and A549 cells. Moreover, the parallel experimental results demonstrated less surficial FITC-labeled Brevinin-1RL1 was observed in Brevinin-1RL1 insensitive noncancer cell lines NCM460 and Beas-2B, indicating Brevinin-1RL1 preferential targeted cancer cells rather than noncancer cells (Figure 5c–f). Aforementioned data suggested that Brevinin-1RL1 might interact with the lipids or particular proteins on the plasma membrane, further inducing apoptosis and necrosis to suppress tumor cells. 

## 3. Discussion

The host defense peptides derived from frog skin secretions are bioactive products with potential antitumor therapeutic activity [8,25,30,31]. In the present study, we described the discovery of an AMP Brevinin-1RL1, which exhibited growth inhibitory effect towards different human tumor cells, detected with the IC_50_ ranging from 5 to 10 μM (Figure 2a). The “Rana box” is a unique structure of peptides and contains an intermolecular disulphide bridge at the C-terminal consisting of seven to nine amino acid residues [32]. Many of these peptides originate from frogs in the Rana genus and examples include Brevinins [33], Esculentins [34], Ranatuerins [35], Ranalexins [36], Gaegurins [37], Nigrocins [38] and Ranacyclins [39]. According to previous studies, the roles of the ‘Rana box’ to the bioactivities of these peptides are controversial [40]. Some studies emphasized the importance of this structure since removing the “Rana box” structure from the peptide greatly reduces the antimicrobial and anti-proliferative activity [41,42,43]. One of the most typical examples for this family of peptides was Brevinin. Neither truncating the C-terminal cyclic nor removin g the “Rana box” structure of Brevinin-1GHa showed lower antimicrobial activity, which may explained by the shorter alpha-helix length of peptide after the removal of the C-terminal cyclic [42]. However, other studies dismissed its role as deletion of disulfide bond or truncating the “Rana box” structure showed no effect on antimicrobial activity, even reducing hemolysis [44,45,46]. Brevivin-1RL1 is an AMP originally isolated from Rana limnocharis composed of 24 amino acids with an intra-molecular disulfide-bridged domain of 7-membered ring located at the C-terminus and the antitumor activity was lost while the disulfide bridge was reduced, indicating the “Rana box” contributed to the bioactivity of Brevivin-1RL1 (Figure 2c). However, whether the “Rana box” of Brevinin-1RL1 involved in the formation α helix structure needs further investigation.

The mechanism of AMPs inhibiting tumor cells is similar to the antimicrobial mechanism but more complicated [12,23]. Membrane lysis is recognized as the consistent mechanism of action between the two [13,14,18,26]. AMPs obtained by natural selection are cationic [47], while the tumor cells have a higher content of anionic phosphatidylserine (PS) and phosphatidylinositol (PI) on their outer leaflets distinguished from noncancer cells [48,49,50]. Thus, AMPs preferentially bind and insert into negatively charged cell membranes specifically by electrostatic attraction to suppress cancers selectively [49]. The typical one is the insect-derived cecropin AMPs, which combine the positive charge on the amphiphilic α-helix with the negative charge on the cell membrane phospholipid molecule and then insert into the cell membrane to cause the loss of cell osmotic pressure and death [13]. In the cell viability assay, Brevinin-1RL1 specifically attenuated the growth of cancer cells, while less cytotoxicity against the immortalized noncancer human cells was observed (Figure 2b). Further cellular localization and binding affinity conducted with FITC-labeled Brevinin-1RL1 confirmed Brevinin-1RL1 preferred aggregated on the surface of cancer cells and exerted anticancer activity (Figure 5a–f). 

Apoptosis is generally regulated by caspases and involves intrinsic mitochondrial pathways and extrinsic death receptor transduction pathways [51]. We showed that Brevinin-1RL1 effectively induced apoptosis in Annexin V+/PI+ double staining (Figure 3a–c) and PI single staining assays (Figure 3d). In addition, both the death receptor pathway and the mitochondrial pathway are involved in Brevinin-1RL1 induced apoptosis, because Brevinin-1RL1 induced the activation of initiator caspase 8 and 9 (Figure 4a,b). The involvement of mitochondrial pathway was further confirmed by loss of mitochondrial membrane potential (Figure 4c,d). Moreover, the involvement of caspases in Brevinin-1RL1-induced cell death was verified by using the pan-caspase inhibitor z-VAD-FMK (Figure 3e). In addition, the release of cell contents and LDH (Figure 3e,f) indicated necrosis was also involved in the cytotoxicity of Brevinin-1RL1 to tumor cells. These results demonstrate that Brevinin-1RL1 induced necrosis and caspase-dependent apoptosis.

In conclusion, our study indicated that the natural derived AMP Brevinin-1RL1 is a potential candidate anticancer agent preferential acted on tumor cells to trigger necrosis and caspase-dependent apoptosis, while the specific target and the therapeutic efficacy on preclinical cancer models still need to be further investigated. Taken together, these findings provided strong evidence to demonstrate that AMP database is an effective resource to develop new cancer therapeutics.

## 4. Materials and Methods

### 4.1. Peptide Synthesis

Brevinin-1RL1 (FFPLIAGLAARFLPKIFCSITKRC, M = 2711.3 g/mol) and a fluorescent dye fluorescein isothiocyanate (FITC) labeled Brevinin-1RL1 (FITC-FFPLIAGLAARFLPKIFCSITKRC, M = 3213.9 g/mol) with an intramolecular disulfide bridge was synthesized by GL Biochem Ltd (Shanghai, China). Purification of the peptides was performed by reversed-phase HPLC (RP-HPLC) on a Vydac 218TP 510 C18 column with a linear gradient of acetonitrile in 0.1% TFA in water at a flow rate of 2 mL/min. Identity of the peptides was confirmed by MALDI-TOF mass spectrometry and protein sequencing. The purity of the synthetic peptides was higher than 95%. The peptide concentrations of the non-labeled peptides were determined via measurement of UV-absorbance of tryptophan at 280 nm (using NanoDrop ND 1000 (Peqlab, VWR International, Inc. Erlangen, Germany)). The peptide and FITC-labeled peptide were dissolved in DMSO to prepare a stock solution (1 mM), stored at −20 °C.

### 4.2. Cell Culture

Human non-small cell lung adenocarcinoma cell line A549, human breast adenocarcinoma cell line MDA-MB-231, human hepatocellular carcinoma cell line SMMC-7721, melanomas cell line B16-F10 and human colorectal adenocarcinoma cell lines HCT116 and SW480 were purchased from Cell Bank of Type Culture Collection of Chinese Academy of Sciences (Shanghai, China). The human normal colon mucosal epithelial cell line NCM460, human bronchial epithelial cell line BEAS-2B and immortalized human keratinocyte cell line HaCaT were purchased from the Shanghai Institute of Biochemistry and Cell Biology, Chinese Academy of Sciences (Shanghai, China). B16-F10, MDA-MB-231,BEAS-2B, SW480 and HaCaT cells were cultured in Dulbecco’s modified Eagle medium (DMEM; Mediatech, Herndon, VA, USA) and A549, SMMC-7721, HCT116 and NCM460 cells were sustained in RPMI-1640 medium (Mediatech, Herndon, VA, USA), supplemented with 10% fetal bovine serum (FBS; Atlanta Biologicals, Lawrenceville, GA, USA), 100 μg/mL streptomycin (HyClone, Logan, UT, USA) and 100 U/mL penicillin (HyClone, Logan, UT, USA) in a humidified atmosphere with 5% CO_2_ according to suppliers’ instructions. All the cells were maintained in a humidified 5% CO_2_ incubator at 37 °C as previously described [52].

### 4.3. Cell Proliferation and Viability Assay

Anticancer activity of Brevinin-1RL1 was determined by MTS (3-(4,5-dimethylthiazol-2-yl)-5-(3-carboxymethoxyphenyl)-2-(4-sulfophenyl)-2H-tetrazolium, inner salt) assay, according to the protocol of CellTiter 96^®^ AQueous One Solution Cell Proliferation Assay kit (Promega, Madison, WI, USA). Briefly, 100 μL of cell suspensions were seeded at 5 × 10^3^ cells/well into each well of 96-well plates and incubate overnight before drug treatment. Then, the cells were either treated with various concentrations of peptides or without peptides for 48 h, followed by 20 μL of MTS per well. After further incubation at 37 °C for 1–3 h, the absorbance was determined using a Wallac 1420 Multilabel Counter (PerkinElmer Life Sciences, Wellesley, MA, USA) by measuring the optical density (OD) at 490 nm. The half inhibitory concentration (IC_50_) was determined by the relative survival curve.

### 4.4. Hemolytic Activity

To determine the toxicity of the peptides to normal mammalian cells, human erythrocytes were used to test the hemolytic activity of the peptides according to the method reported [53]. Blood was collected from the median cubital vein of healthy volunteers. The normal human erythrocytes were washed three times with Dulbecco’s phosphate-buffered saline (PBS; Biological Industries, Israel), with the resulting pellet was suspended in PBS and diluted to 5% (*w*/*v*). The sample peptide was diluted with physiological saline into various gradient concentrations, 200 μL of various gradient concentrations of samples and 200 μL diluted red blood cells were gentle mixed and incubated at 37 °C for 30 min. Parallel incubations of erythrocytes in the presence of normal saline or 1% Triton X-100 served as the negative and positive controls, respectively. After incubation, centrifugation was conducted at 2500 rpm/min for 5 min at room temperature. A total of 100 μL of the supernatant was taken to measure the absorbance at 540 nm (GeneQuant, Fairfield, CT, USA). The relative optical density was compared with the absorbance of 1% Triton X-100, which induce 100% hemolysis. The study was proved by the Research Ethics Committee of Kunming Institute of Botany, Chinese Academy of Sciences and the ethics approval number was Kib202103028. Informed consent was provided for blood donation. 

### 4.5. Cell Morphological Analysis

A549 and HCT116 cells were seeded (1 × 10^5^ cells/well) in complete culture medium. The next day, the cells were treated with Brevinin-1RL1 (0, 6 and 12 μM) for 24 h, images were taken with an Olympus inverted phasecontrast microscope (Olympus Optical Co., Melville, NY, USA) (200×) equipped with the Quick Imaging system.

### 4.6. Lactate Dehydrogenase (LDH)-Release Assay

The LDH release assay was performed by the CytoTox 96 non-radioactive cytotoxicity assay kit (Promega; Charbonnie’res-les-Bains, France) according to the manufacturer’s protocol as previously described [54]. Briefly, cells seeded in 96-well plates (5 × 10^3^ cells/well/100 μL) were incubated with various concentrations of Brevinin-1RL1 or equivalent DMSO for 24 h. Then, cells were lysed for 45 min served as the spontaneous and maximum LDH activity controls for 45 min before running the assay. After centrifugation of the microtiter plates, 50 μL of supernatant was transferred into new tube from each well and mixed with equal volume of supplied reaction mixture and incubated at 37 °C for 30 min. A total of 50 μL of supplied stop solution was added to each well and the absorbance of the colored formazan was determined using the microplate reader (Perkin-Elmer Life Sciences, Waltham, MA, USA) at 450 nm wavelength. Cell membrane integrity was evaluated by measuring the LDH activity released and the assay was repeated in triplicate.

### 4.7. Analysis of Cell Cycle 

Cells were seeded in 6-well plates at a density of 2 × 10^5^ cells/well and treated with various concentrations of the peptide for 24 h. Cells were collected and fixed with pre-cold 70% ethanol overnight at −20 °C. After the fixation, the cells were collected and washed twice with precooled PBS, then 50 mg/mL RNase A (Sigma-Aldrich) was added for digestion at 37 °C for 30 min. Then, 50 mg/mL Propidium iodide (PI) was added to each sample for 15 min at room temperature in dark. The fluorescent intensity was evaluated by flow cytometric analysis with FACS Calibur flow cytometer (Becton Dickinson, San Jose, CA). 

### 4.8. Analysis of Cell Apoptosis 

Cell apoptosis was analyzed by the Annexin V-FITC/PI Apoptosis kit (BD Biosciences, New Jersey, USA) according to the manufacturer’s protocol. Cells were seeded in 6-well plates at a density of 1 × 10^5^ cells/well and kept for 24 h, then treated with Brevinin-1RL1 for 48 h. Cells were collected and washed twice with cold PBS and then resuspended in 100 μL of Annexin-V binding buffer, followed by incubation with Annexin V-FITC and Propidium iodide (PI) for 15 min at room temperature in dark. The fluorescent intensity was evaluated by flow cytometric analysis with FACS Calibur flow cytometer (Becton Dickinson, San Jose, CA). Cells treated with vehicle alone (DMSO) were used as a control.

### 4.9. Mitochondrial Membrane Potential

The mitochondrial membrane potential was performed by the JC-1 Mitochondrial Membrane Potential Assay Kit (Cayman Chemical; Michigan, MI, USA) according to the manufacturer’s protocol. JC-1 is an ideal fluorescent probe widely used to detect the △Ψm position of mitochondrial membrane electricity. Cells seeded in 6-well plates (2 × 10^5^ cells) were exposed to Brevinin-1RL1 or equivalent DMSO for 24 h. Then, 200 μL JC-1 staining working solution were added to each sample in the cultured cells and incubated at 37 °C for 30 min in the dark. Then, collected the cells into a flow tube and analyze the fluorescence intensity with a flow cytometer.

### 4.10. Immunoblotting Analysis

Immunoblotting analysis was performed as described previously [55]. Cells were seeded in 6-well plates with a density of 3 × 10^5^ cells/well and treated with or without various concentrations of Brevinin-1RL1 for 24 h. Then, the cells were harvested and lysed in RIPA buffer (50 mM Tris-HCl [pH 7.4], 150 mM NaCl, 1% sodium deoxycholate, 0.1% sodium dodecyl sulfate, 1% NP-40, 1 mM EDTA and 1 mM PMSF) that supplemented with protease inhibitors and phosphatase inhibitors (Roche). The lysate was shaken and centrifuged, the supernatant was extracted and quantified with the BCA kit (Thermo Scientific, Waltham, MA, USA) and then the loading buffer was added and boiled for 5 min. Protein extracts were separated by denaturing SDS-PAGE and transferred to polyvinylidene fluoride (PVDF) membranes (Millipore). Membranes were blocked with 5% non-fat milk in TBST and incubated overnight at 4 °C with the following primary antibodies: anti-Caspase-9, anti-Cleaved Caspase-3 and anti-Cleaved PARP (Cell Signaling Technology, Beverly, MA, USA); anti-Caspase-3, anti-Caspase-8, anti-Cleaved Caspase-8 and anti-Actin (Santa Cruz Technology, Dallas, TX, USA). The membrane was washed three times with PBST and then incubated with corresponding secondary antibody conjugated to horseradish peroxidase for 2 h at room temperature. Target protein band were incubated with Pierce ECL substrate and visualized by chemiluminescent detection on an ImageQuant LAS 4000 mini (GE Healthcare, Pittsburgh, Pittsburgh, PA, USA).

### 4.11. Confocal Fluorescence Microscopy

HCT116, NCM460, BEAS-2B and A549 cells were seeded and cultured in glass-bottom 6-well plates with a density of 1 × 10^5^ cells/well overnight and then incubated with FITC-labeled Brevinin-1RL1 or buffer alone for 12 h. After treatment, cells were fixed with 4% paraformaldehyde for 20 min and were permeabilized with 0.1% Triton X-100 for 10 min. Then, 4′, 6-diamidino-2-phenylindole (DAPI) was employed to stain the nuclei. Microscopic analysis was traced the FITC-labeled peptides with laser confocal scanning microscope.

### 4.12. TEM Assay

Briefly, A549 and HCT116 cells were seeded at a density of 5 × 10^4^ cells/dish in 60 mm dishes. The next day, the cells were incubated with or without Brevinin-1RL1 for 24 h. After treatment, the cells were washed twice with PBS and followed by an overnight fixation with 2% glutaraldehyde in 0.1 M Sorensen phosphate buffer (pH 7.4) at 4 °C overnight. Then, the samples were washed and fixed in 1% osmium tetroxide for 1 h at room temperature. Samples were then dehydrated in a series of graded ethanol solutions followed by propylene oxide and cells were embedded in Epon 812 resin, which was then cut into 1 mm^3^ blocks. The subsequent samples were cut with ninety nanometer-thick sections and doubly contrasted with uranyl acetate and lead citrate for TEM analysis (JEOL) at an accelerating voltage of 80 kV.

### 4.13. Statistical Analysis

All statistical analyses were performed using the GraphPad PrismTM version 6.02 software from GraphPad Software Inc. (San Diego, CA, USA). In addition, the data are presented as the mean values standard deviation (SD) and statistical differences were analyzed by one-way and two-way ANOVA followed by Tuckey’s post hoc test using the software package SPSS 11. The results were conducted at least three determinations for each test from three independent experiments. The statistical significance of the differences is given as * p < 0.05; ** p < 0.01; *** p < 0.001. 

## Figures and Tables

**Table 1:** Amino acid sequence, length, molecular weight and related biological parameters of Brevinin-1RL1.

Peptide	Sequence	Length (a.a)	MW (Da)	Net Charge	pI	Hydropho-bicity (H)	Freq Polar
Brevinin-1RL1	FFPLIAGLAARFLPKIFCSITKRC	24	2711.3	4	10.11	0.805	0.292

Notes: Molecular weight and isoelectric point (pI) of Brevinin-1RL1 were calculated by http://web.expasy (accessed on: 18 December 2019). Org/computer/. The mean Hydrophobicity (H) and Freq Polar of Brevinin-1RL1 were estimated by http://heliquest.ipmc.cnrs.fr/ (accessed on: 12 July 2020).

**Table 2:** The IC_50_ of Brevinin-1RL1 against different cells.

Cell Type Caption	Cell Lines	Tumor Type	IC_50_ (Mean ± SD, μM)
Tumor cells	HCT116	colorectal adenocarcinoma	5.87 ± 0.15
	MDA-MB-231	breast adenocarcinoma	5.44 ± 0.33
	SW480	colorectal adenocarcinoma	10.37 ± 0.40
	A549	lung adenocarcinoma	5.81 ± 0.23
	SMMC-7721	hepatocellular carcinoma	6.87 ± 0.51
	B16-F10	melanomas	6.65 ± 0.33
Immortalized noncancer cells	NCM460	colon mucosal epithelial	16.84 ± 0.56
	BEAS-2B	bronchial epithelial	16.57 ± 0.29
	HaCaT	keratinocyte cell	28.67 ± 0.36

## Data Availability

The data presented in this study, are available in this article.

## Supplementary Materials

The following are available online. Figure S1: The purity and mass spectrometry and structure of FITC-labeled Brevinin-1RL1. (a) The HPLC chromatogram of FITC-labeled Brevinin-1RL1. (b) The MS of FITC-labeled Brevinin-1RL1.

## Sample Availability

Samples of the compounds are available from the authors.
