# Supplementary material for: Antimicrobial Peptide Brevivin-1RL1 from Frog Skin Secretion Induces Apoptosis and Necrosis of Tumor Cells
Source: Molecules. 2021 Apr 3;26(7):2059. doi: 10.3390/molecules26072059 (PMC8038347; doi:10.3390/molecules26072059)
Supplement: Supplementary file 1 [file molecules-26-02059-s001.pdf]

(a)

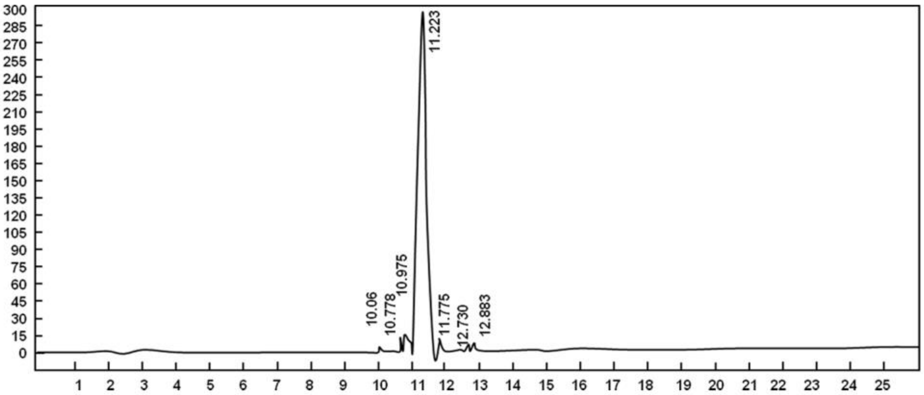

Peak No.	Ret Time	Height	Area	Conc..
1	10.060	594.278	10848.711	0.2785
2	10.778	1846.288	10787.567	0.2770
3	10.975	8701.301	96942.125	2.4891
4	11.223	282047.688	3724079.000	95.6188
5	11.775	1425.880	15216.490	0.3907
6	12.730	1779.512	21542.646	0.5531
7	12.883	1621.198	15295.846	0.3927
Total				100.0000

(b)

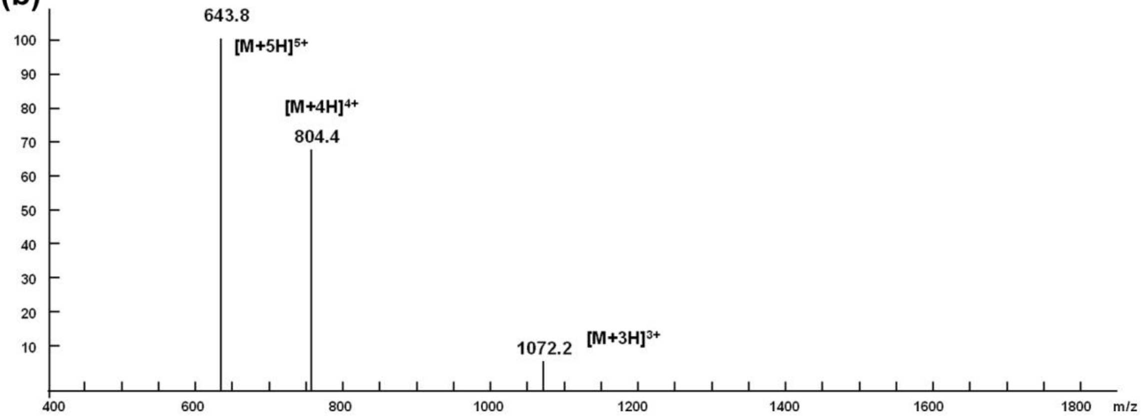

Figure S1: The purity and mass spectrometry and structure of FITC-labeled Brevinin-1RL1. (**a**) The HPLC chromatogram of FITC-labeled Brevinin-1RL1. (**b**) The MS of FITC-labeled Brevinin-1RL1.
